# Intrinsic bevacizumab resistance is associated with prolonged activation of autocrine VEGF signaling and hypoxia tolerance in colorectal cancer cells and can be overcome by nintedanib, a small molecule angiokinase inhibitor

**DOI:** 10.18632/oncotarget.1671

**Published:** 2014-01-20

**Authors:** Paul Mésange, Virginie Poindessous, Michèle Sabbah, Alexandre E. Escargueil, Aimery de Gramont, Annette K. Larsen

**Affiliations:** ^1^ Cancer Biology and Therapeutics, Centre de Recherche Saint-Antoine; ^2^ Institut National de la Santé et de la Recherche Médicale U938, Paris, France; ^3^ Université Pierre et Marie Curie, Paris, France; ^4^ Universite Paris Descartes, Paris, France; ^5^ Department of Medical Oncology, Hôpital Saint-Antoine, Paris, France

**Keywords:** resistance, angiogenesis inhibition, vascular endothelial growth factor (VEGF)-signaling, hypoxia, bevacizumab, ninte-danib

## Abstract

Colorectal cancer (CRC) is a common tumor type with a high mortality rate, in part due to intrinsic drug resistance. Although bevacizumab, a VEGF-directed neutralizing antibody, is particularly active in this pathology, some patients never respond for reasons not well understood. We here wish to clarify the role of autocrine VEGF signaling in the response of CRC cells to angiogenesis inhibition. Our results show that CRC cells with intrinsic bevacizumab-resistance displayed pronounced upregulation of autocrine HIF-VEGF-VEGFR signaling in response to prolonged bevacizumab exposure whereas the same signaling pathway was downregulated in bevacizumab-sensitive xenografts. Importantly, both bevacizumab-sensitive and -resistant CRC xenografts were sensitive to nintedanib, a small molecule angiokinase inhibitor, which was associated with inhibition of mTORC1. *In vitro* studies revealed that bevacizumab-resistant cells displayed intrinsically higher HIF-VEGF signaling intensity and hypoxia tolerance compared to their bevacizumab-sensitive counterparts. Interestingly, although nintedanib showed comparable activity toward bevacizumab-sensitive cells under normoxia and hypoxia, the drug was three-fold more toxic to the resistant cells under hypoxia, suggesting that nintedanib attenuated the survival signaling that usually protects these cells from hypoxia-mediated cell death. In conclusion, our findings support a role for autocrine VEGF signaling in the survival of CRC cells to hypoxia and thus to angiogenesis inhibition. We further show that nintedanib, a small molecule angiokinase inhibitor, is active toward CRC models with intrinsic bevacizumab resistance supporting clinical trials of nintedanib in patients that do not respond to bevacizumab, alone or in combination with bevacizumab to increase angiogenesis inhibition.

## INTRODUCTION

Neovascularization (sprouting angiogenesis) is required for the growth of most solid tumors and facilitates the spread of tumor cells to secondary sites [1] providing a rational basis for the clinical use of angiogenesis inhibitors. Vascular endothelial growth factor A (hereafter referred to as VEGF) is a key regulator of angiogenesis. Amplification of the VEGF locus is observed in a subset of patients with metastatic colorectal cancer (mCRC) and is associated with a remarkably aggressive disease characterized by a high incidence of vascular invasion [2]. Recent data from more than 2000 mCRC patients indicate that high baseline plasma levels of VEGF might be a negative prognostic factor for both progression-free survival (PFS) and overall survival (OS) [3]. Therefore, VEGF signaling is linked to invasiveness and aggressive disease in CRC and appears as an attractive therapeutic target.

Several VEGF(R)-targeted agents are approved or are undergoing clinical trials for treatment of CRC. Bevacizumab (avastin), a VEGF-neutralizing monoclonal antibody [4], was the first angiogenesis inhibitor to be approved and represents the current benchmark. Although bevacizumab shows excellent activity in some patients, others never respond, for reasons not well understood. Nintedanib (BIBF 1120) is a small molecule tyrosine kinase inhibitor that inhibits several angiokinases including VEGFR1/Flt1, VEGFR2/Flk1/KDR and VEGFR3/Flt4 as well as FGFR1, FGFR2, FGFR3, PDGFR-alpha, PDGFR-beta and Flt3 [5]. Nintedanib is currently in phase III clinical trials in ovarian and non-small cell lung cancer (NSCLC), where it has been successful in combination with taxotere [6].

Although the mechanism of angiogenesis inhibitors is not fully elucidated, it is generally believed that pruning of the tumor microvasculature will decrease blood supply thereby diminishing tumor oxygenation and promoting tumor cell death [7-11]. Hypoxia is accompanied by activation of the hypoxia-inducible transcription factors HIF-1alpha and HIF-2alpha leading to increased expression of VEGF and other HIF targets [12, 13]. Binding of VEGF to its receptors triggers signaling pathways that modulate the phosphorylation, stability and/or activity of a variety of down-stream targets including HIF and VEGF [14-17], thereby initiating a positive feed-back loop. Thus, HIF-VEGF signaling intensity emerges as a key factor in determining the outcome of angiogenesis inhibition.

A striking feature of CRC is the capacity for both paracrine and autocrine VEGF-signaling. CRC cells are a major source of VEGF that interacts with VEGF receptors on tumor-associated endothelial cells thereby stimulating their growth, migration and survival [18]. In addition, CRC cells express VEGF receptors (VEGFRs) giving rise to autocrine VEGF signaling [19]. Results from different laboratories indicate that most, if not all, human CRC cells and tumors express functional VEGFR1 [20-23] as well as VEGFR2 [24-26]. Autocrine VEGF signaling promotes CRC survival under different types of stress including 5-fluorouracil exposure, low serum conditions and anchorage-independent growth [27-29]. Interestingly, VEGFR1-mediated VEGF signaling may be particularly important for the survival of invasive CRC cells that have undergone the epithelial-mesenchymal transition and no longer benefit from homotypic cell-cell contact [20].

Until now, most studies on both intrinsic and acquired bevacizumab resistance have focused on paracrine VEGF signaling and tumor-associated endothelial cells. However, the capacity of autocrine VEGF-signaling to promote CRC cell survival under different types of stress suggests that this pathway may also play a role during angiogenesis inhibition.

We now report that bevacizumab-resistant, but not bevacizumab-sensitive, CRC cells showed strong autocrine HIF-VEGF-VEGFR signaling in response to prolonged bevacizumab exposure *in vivo* and displayed intrinsically higher HIF-VEGF signaling intensity and hypoxia tolerance *in vitro*. We further show that tumors with intrinsic bevacizumab resistance remain sensitive to nintedanib, a small molecule angiokinase inhibitor. These data suggest that the antitumor activity of at least some small molecule angiokinase inhibitors is not limited by the mechanisms underlying natural bevacizumab resistance and provide a rational for clinical trials of nintedanib in CRC patients that do not respond to bevacizumab, alone or in combination with bevacizumab to increase angiogenesis inhibition.

## RESULTS

### HT-29 xenografts are naturally bevacizumab-resistant but remain sensitive to nintedanib, a small molecule angiokinase inhibitor

**Figure 1:**
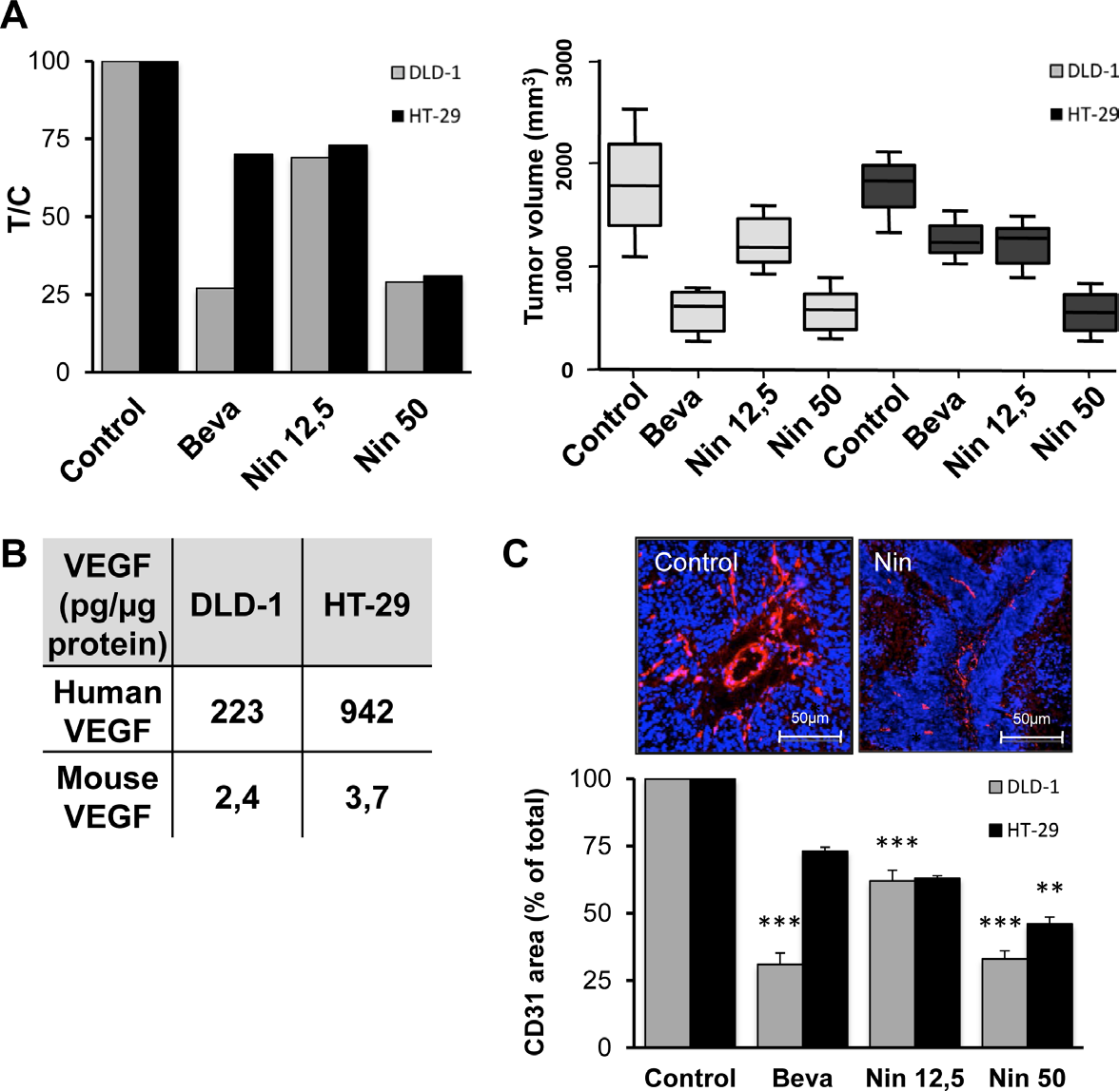
Influence of bevacizumab and nintedanib on tumor growth and angiogenesis in CRC xenografts (A) Nude mice with DLD-1 (grey columns) or HT-29 (black columns) human CRC xenografts were dosed with vehicle (Control), bevacizumab at 5 mg/kg i.p. every 3 days (Beva), nintedanib at 12,5 mg/kg p.o. once daily (Nin 12,5) or nintedanib at 50 mg/kg p.o. once daily (Nin 50) for 4 weeks. Each treatment group corresponded to at least seven animals. Left, T/C values were determined as follows: average tumor volume of treated animals/average tumor volume of the vehicle controls x100. Right, box and whisker plot of the tumor volumes of DLD-1 (light grey boxes) or HT-29 (dark grey boxes) xenografts after 4 weeks treatment with bevacizumab or nintedanib. Lines, medians; boxes, 25th to 75th percentile interquartile ranges; whiskers, the highest and lowest values for a given treatment. (B) Total protein was extracted from DLD-1 and HT-29 xenografts (pool of 3 tumors per xenograft model) and the amounts of human (tumor-derived) and murine (stroma-derived) VEGF were determined by ELISA. (C) Top, representative images of xenografts from animals treated with vehicle or with nintedanib at 50 mg/kg p.o. once daily. CD31-positive blood vessels are outlined in red whereas the nuclei appear in blue. Bottom, quantitative image analysis of the CD31 signal for DLD-1 (grey columns) and HT-29 tumors (black columns). The data show the CD31-positive area, as % of total, and represent the average of at least 6 fields/tumor for at least 3 different tumors. The statistical analysis of experimental data was performed using a Student's paired t-test comparing the treatment group with the vehicle control. Bars, SD; * p < 0,05; ** p < 0,01; *** p < 0,001.

Continued bevacizumab treatment of mice carrying human CRC xenografts revealed that HT-29 tumors are naturally bevacizumab-resistant with only 29% growth inhibition after 4 weeks drug exposure while the same scheduling resulted in 68% tumor growth inhibition for the bevacizumab-sensitive DLD-1 tumors (Figure 1A).

To establish if bevacizumab resistance is associated with cross-resistance to small molecules targeting the same pathways, nintedanib was administered to animals with DLD-1 or HT-29 xenografts. The results show that nintedanib displays comparable tumor growth inhibitory activity in the two tumor models (Figure 1A). No weight loss or other toxic side effects were noted for either bevacizumab or nintedanib (data not shown). Therefore, CRC tumors with intrinsic bevacizumab resistance remain sensitive to at least some small molecule angiokinase inhibitors.

### Bevacizumab-resistant xenografts express high levels of VEGF

Since bevacizumab and nintedanib both interfere with VEGF signaling, tissue extracts were prepared from untreated DLD-1 and HT-29 tumors and the concentration of human VEGF was determined by ELISA analysis (Figure 1B). The amounts of VEGF were at least 4 times higher in tumor tissues from HT-29 xenografts compared to DLD-1 xenografts (942 pg vs. 223 pg VEGF/μg protein, respectively, p < 0,001).

Bevacizumab is specific for human VEGF, and will therefore only neutralize VEGF produced by the tumor cells, but not murine VEGF secreted by the tumor-associated stromal cells. To evaluate the contribution of stromal VEGF, tissue extracts were prepared from untreated DLD-1 and HT-29 tumors and the concentration of murine VEGF was determined by ELISA analysis. The results show that murine VEGF represents a minor fraction of the total VEGF (0,4 to 1.1%) in both tumor models (Figure 1B). Therefore, VEGF signaling is principally mediated by human, tumor-derived VEGF in both DLD-1 and HT-29 xenografts.

### Bevacizumab has limited activity on the microvasculature in bevacizumab-resistant xenografts

The microvascular density of DLD-1 and HT-29 xenografts was compared by IHC with a CD31-directed antibody followed by quantitative image analysis. The results (Figure 1C) indicate that bevacizumab reduces the microvascular density by 70% in DLD-1 xenografts, compared to the vehicle control. In contrast, the same schedule of bevacizumab only reduced the microvascular density by 27% in HT-29 xenografts. These results show that the microvascular density was less influenced by bevacizumab in the bevacizumab-resistant xenografts whereas nintedanib had comparable activity in the two models.

### Bevacizumab and nintedanib show a combination of cytostatic and cytotoxic activities

**Figure 2:**
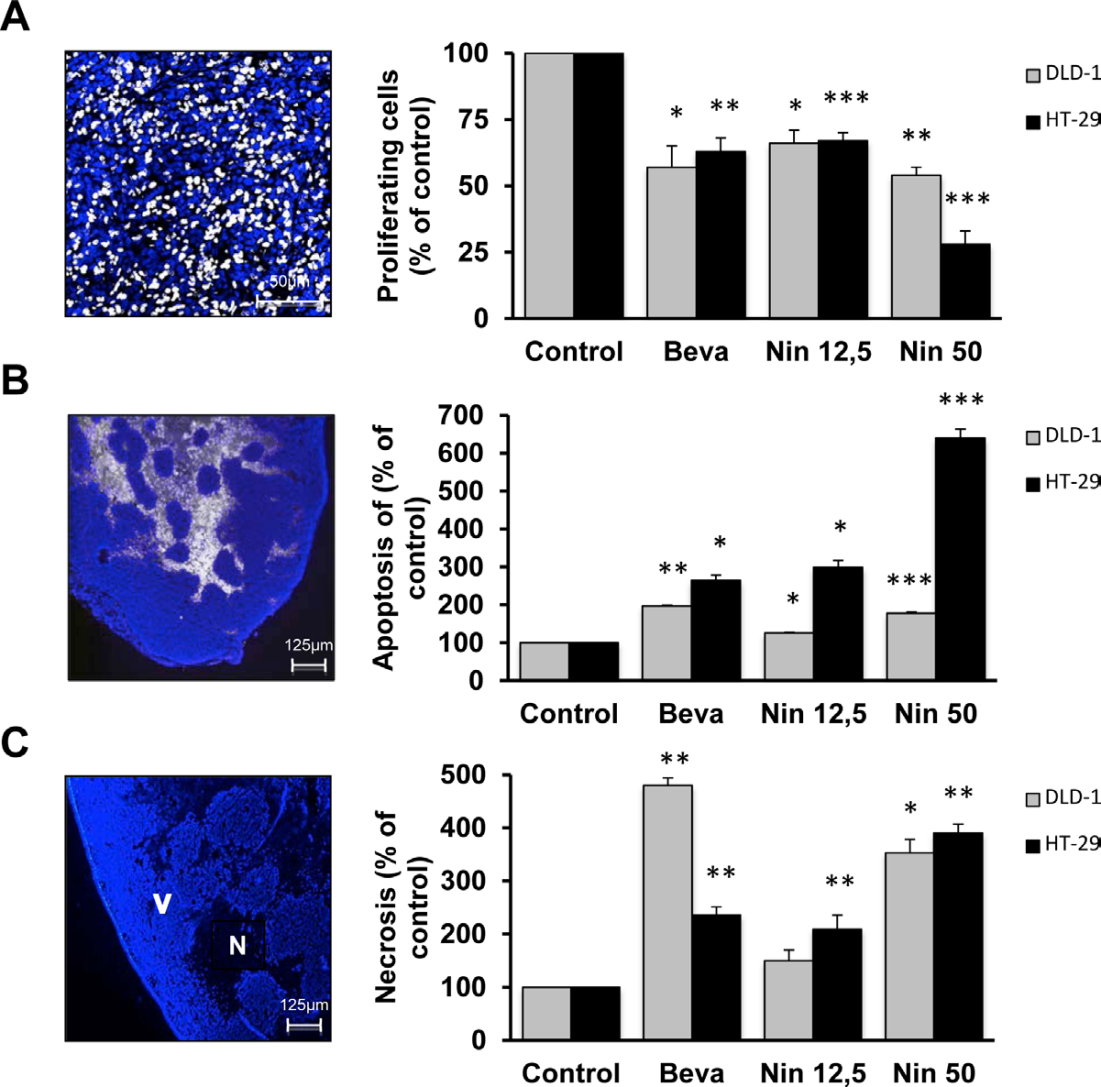
Cytostatic and cytotoxic effects of bevacizumab and nintedanib in CRC xenografts Influence of bevacizumab and nintedanib on (A) DNA synthesis, (B) apoptosis and (C) necrosis in DLD-1 (grey columns) and HT-29 (black columns) tumors. Animals with CRC xenografts were dosed with vehicle (Control), bevacizumab at 5 mg/kg i.p. every 3 days (Beva), nintedanib at 12,5 mg/kg p.o. once daily (Nin 12,5) or nintedanib at 50 mg/kg p.o. once daily (Nin 50) for 4 weeks. The photos illustrate typical staining patterns. V, viable tissue; N, necrotic tissue. DNA synthesis is determined as the ratio between EdU-positive cells and the total number of viable cells and corresponds to the average of 6 fields/tumor (each field representing approximately 1700 cells) from at least 3 different tumors. Apoptosis is expressed as the area of TUNEL-positive cells, as % of the total area of viable cells, and is the average of 6 fields/tumor for at least 3 different tumors. For necrosis, the data indicates the surface of necrotic cells as % of the total surface and is the average of 6 fields/tumor for at least 3 different tumors. The statistical analysis of experimental data was performed using a Student's paired t-test comparing the treatment group with the vehicle control. Bars, SD; * p < 0,05; ** p < 0,01; *** p < 0,001.

Tumor growth inhibition may be due to both cytostatic (cell cycle arrest) and cytotoxic (cell death) effects. We therefore evaluated the influence of bevacizumab and nintedanib on *in vivo* DNA synthesis (EdU incorporation), apoptotic cell death (TUNEL assay) and necrotic cell death (Figure 2).

We first compared the influence of the two drugs. In DLD-1 xenografts, bevacizumab and nintedanib (50 mg/kg) have comparable antitumor activity. Both agents displayed a mixture of cytotostatic and cytotoxic effects without any striking differences between them: 43% vs 46% decrease in DNA synthesis as determined by EdU, 197% vs 178% increase in apoptotic cell death as determined by TUNEL, and 480% vs 353% increased necrosis for bevacizumab and nintedanib, respectively. (Figure 2). In HT-29 xenografts, bevacizumab and low dose nintedanib have comparable antitumor activity. Both agents displayed a mixture of cytostatic and cytotoxic effects without any striking differences between them: 37% vs 33% decrease in DNA synthesis, 265% vs 299% increased apoptosis, and 236% vs 209% increased necrosis for bevacizumab and nintedanib, respectively (Figure 2).

We then compared the influence of nintedanib in the two tumor models. Interestingly, both doses of nintedanib induced more apoptosis in HT-29 tumors (299% and 640%, for the low and high dose of nintedanib, respectively) than in DLD-1 tumors (126% and 178%, for the low and high dose of nintedanib, respectively), whereas the induction of necrosis was comparable (Figure 2).

Taken together, our findings indicate that treatment with both bevacizumab and nintedanib resulted in a mixture of cytostatic and cytotoxic effects. The pronounced apoptosis in nintedanib-treated HT-29 tumors suggests that nintedanib may not only display antivascular effects *in vivo* but also be able to interfere with survival signaling in the CRC cells.

### Angiogenesis inhibition has markedly different influence on HIF-VEGF signaling in bevacizumab sensitive and -resistant tumors

**Figure 3:**
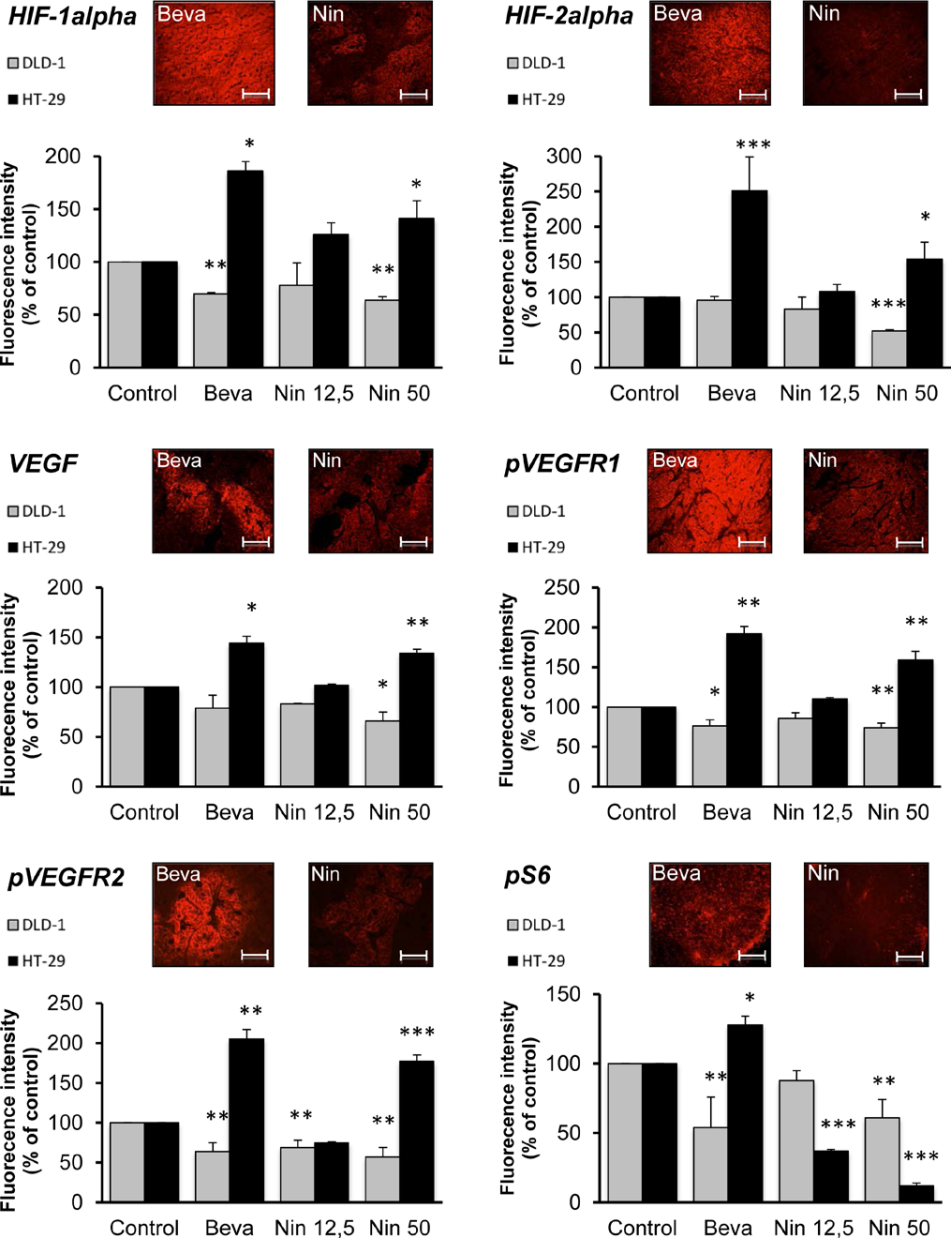
Influence of bevacizumab and nintedanib on HIF-VEGF-VEGFR signaling in CRC xenografts Nude mice with DLD-1 (grey columns) or HT-29 (black columns) human CRC xenografts were dosed with vehicle (Control), bevacizumab at 5 mg/kg i.p. every 3 days (Beva), nintedanib at 12,5 mg/kg p.o. once daily (Nin 12,5) or nintedanib at 50 mg/kg p.o. once daily (Nin 50) for 4 weeks. The expression of HIF-1alpha, HIF-2alpha, VEGF, pVEGFR1, pVEGFR2 and pS6 was determined by immunohistochemistry followed by quantitative image analysis. The photos illustrate the typical staining patterns for tumors derived from animals treated with bevacizumab (Beva) or nintedanib (Nin, 12,5 mg/kg) that provided comparable tumor growth inhibition. For the quantitative analysis of the signal intensity, the data represent the average fluorescence intensity of treated tumors compared to the treatment intensity of control tumors and are the average of 6 fields/tumor for at least 3 different tumors. The statistical analysis of experimental data was performed using a Student's paired t-test comparing the treatment group with the vehicle control. Bars, SD; * p < 0,05; ** p < 0,01; *** p < 0,001.

Microvascular pruning limits oxygen supply to the tumor thereby activating the hypoxia-inducible transcription factors, HIF-1alpha and HIF-2alpha. This is accompanied by transcriptional upregulation of HIF targets like VEGF, thereby promoting autocrine VEGF signaling. The presence of HIF-1alpha, HIF-2alpha, VEGF and the active autophosphorylated forms of VEGFR1 and VEGFR2 in the two xenograft models was revealed by IHC followed by quantitative image analysis (Figure 3). The results revealed striking differences between DLD-1 and HT-29 xenografts, since all treatments were accompanied by attenuation of the HIF-VEGF-VEGFR axis in DLD-1 tumors, but activation of the same signaling components in HT-29 xenografts.

For DLD-1 tumors, there were no marked differences between nintedanib (50 mg/kg) and bevacizumab, two treatments with comparable antitumor activity, for most markers (Figure 3), although the downregulation of HIF-2alpha was more marked for nintedanib-treated tumors than for bevacizumab-treated tumors. In clear contrast, for HT-29 tumors, the signal for all markers was up to 3-fold higher following bevacizumab exposure than after exposure to nintedanib (12,5 mg/kg), in spite of their comparable antitumor and antivascular activities. In particular, for the nintedanib- and bevacizumab-treated tumors, the signal for pVEGFR1 was 110% vs 192% while the signal for pVEGFR2 was 75% vs 205%, respectively. These findings likely reflect a direct inhibitory effect of nintedanib on the tumor cell-associated VEGF receptors, consistent with the potent angiokinase inhibitory activity of this compound.

Taken together, these results reveal both tumor- and drug-specific differences with respect to HIF-VEGF-VEGFR signaling. For the bevacizumab-sensitive DLD-1 tumors, all treatments were accompanied by downregulation of the HIF-VEGF-VEGFR axis whereas the same treatments resulted in activation of autocrine VEGF signaling in the bevacizumab-resistant HT-29 tumors. Furthermore, for HT-29 tumors, bevacizumab treatment was associated with much stronger activation of autocrine VEGF signaling than was the case for nintedanib.

### Nintedanib, but not bevacizumab, attenuates mTORC1 activity in bevacizumab-resistant xenografts

VEGF receptors and other RTKs mediate multiple downstream signaling pathways that may be integrated at the level of mTORC1, the mammalian target of rapamycin (mTOR) complex 1 [30]. Recent findings suggest that inhibition of mTORC1 activity, as measured by tumor levels of Ser240/Ser244-phosphorylated S6 (pS6) is a robust biomarker for the antitumor activity of at least some protein kinase inhibitors such as inhibitors of mutant BRAF and PI3K [31, 32]. In the present study, all treatments reduced the expression of pS6 in DLD-1 tumors, which was most pronounced for bevacizumab and nintedanib at the 50 mg/kg dose (Figure 3). In HT-29 tumors, nintedanib induced a marked, dose-dependent decrease of pS6 levels reaching 12% for the high dose of nintedanib, compared to the vehicle control. In clear contrast, bevacizumab increased the levels of pS6 to ~130% in the same xenograft model.

### Bevacizumab and nintedanib show synergistic activity toward bevacizumab-resistant xenografts

**Figure 4:**
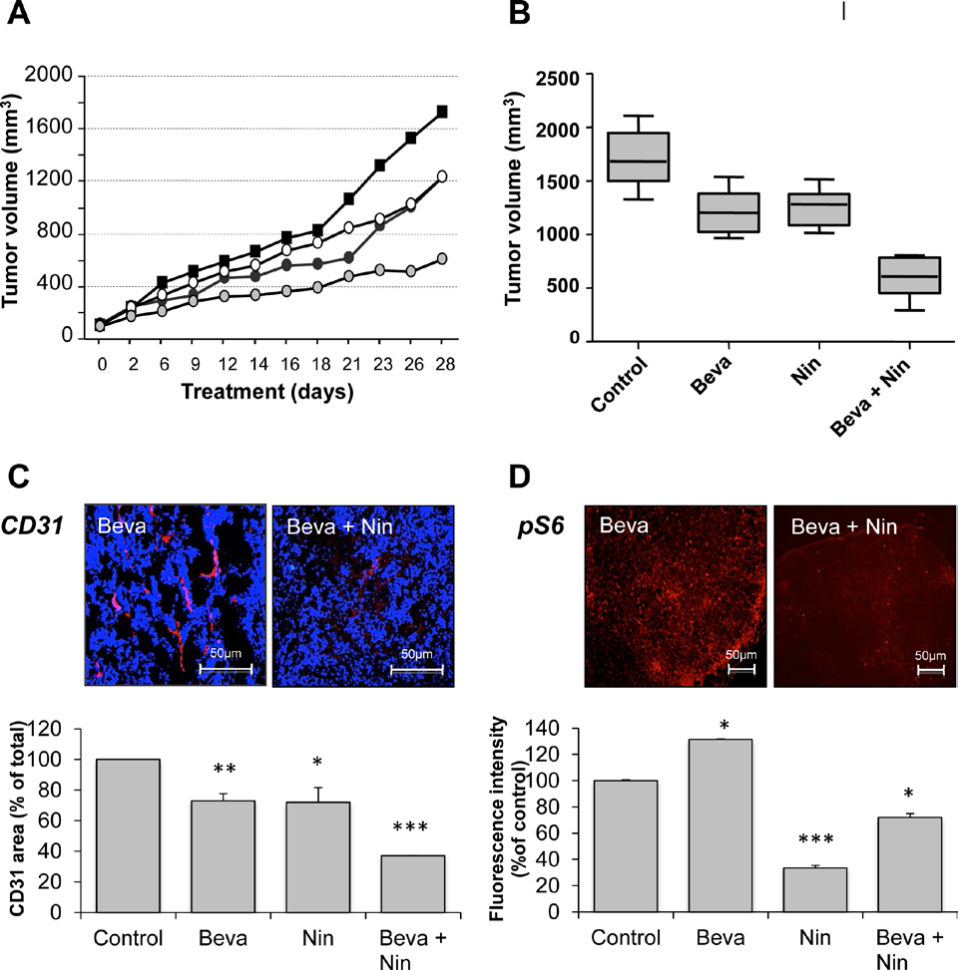
Influence of bevacizumab, nintedanib and their combination on tumor growth, microvessel density and mTORC1 activity in bevacizumab-resistant xenografts Nude mice with HT-29 human CRC xenografts were dosed with vehicle (Control), bevacizumab at 5 mg/kg i.p. every 3 days (Beva), nintedanib at 12,5 mg/kg p.o. once daily (Nin 12,5) or with a combination of bevacizumab and nintedanib for 4 weeks. Each treatment group corresponded to at least seven animals. (A) Average tumor growth of HT-29 tumorgrafts in mice treated with vehicle (black squares), bevacizumab (black circles), nintedanib (white circles) or their combination (light grey circles). (B) Box and whisker plot of the tumor volumes of HT-29 xenografts after 4 weeks treatment with bevacizumab, nintedanib or their combination. Lines, medians; boxes, 25th to 75th percentile interquartile ranges; whiskers, the highest and lowest values for a given treatment. The expression of CD31 (C) and pS6 (D) was determined by immunohistochemistry followed by quantitative image analysis. The photos illustrate typical staining patterns for tumors derived from animals treated with bevacizumab or with a combination of bevacizumab plus nintedanib. For the quantitative analysis of the signal intensity, the data represent the average fluorescence intensity of treated tumors compared to the treatment intensity of control tumors and are the averages of 6 fields/tumor for at least 3 different tumors. The statistical analysis of experimental data was performed using a Student's paired t-test comparing the treatment group with the vehicle control. Bars, SD; * p < 0,05; ** p < 0,01; *** p < 0,001.

The important differences in the activities of bevacizumab and nintedanib toward HT-29 xenografts prompted us to explore the combination of the two agents (Figure 4). Bevacizumab and low dose nintedanib showed comparable activity toward HT-29 xenografts as single agents with approximately 27% tumor growth inhibition. In comparison, the combination of bevacizumab and nintedanib was accompanied by 65% tumor growth inhibition (p < 0,001, bevacizumab alone vs bevacizumab and nintedanib). The combination was active toward both endothelial and tumor cells. The microvascular density, as determined by CD31 staining, decreased by ~28% when bevacizumab or nintedanib were given alone, to 63% when the two agents were given together (p < 0,001, bevacizumab alone vs bevacizumab and nintedanib). In addition, nintedanib was able to counteract the activation of mTORC1 in the tumor cells, as measured by pS6, from 131% (compared to the vehicle control) when bevacizumab was given alone to 72% when the two agents were given together (p < 0,001, bevacizumab alone vs bevacizumab and nintedanib).

### Brief hypoxia is accompanied by HIF-1alpha expression in both DLD-1 and HT-29 cells

**Figure 5:**
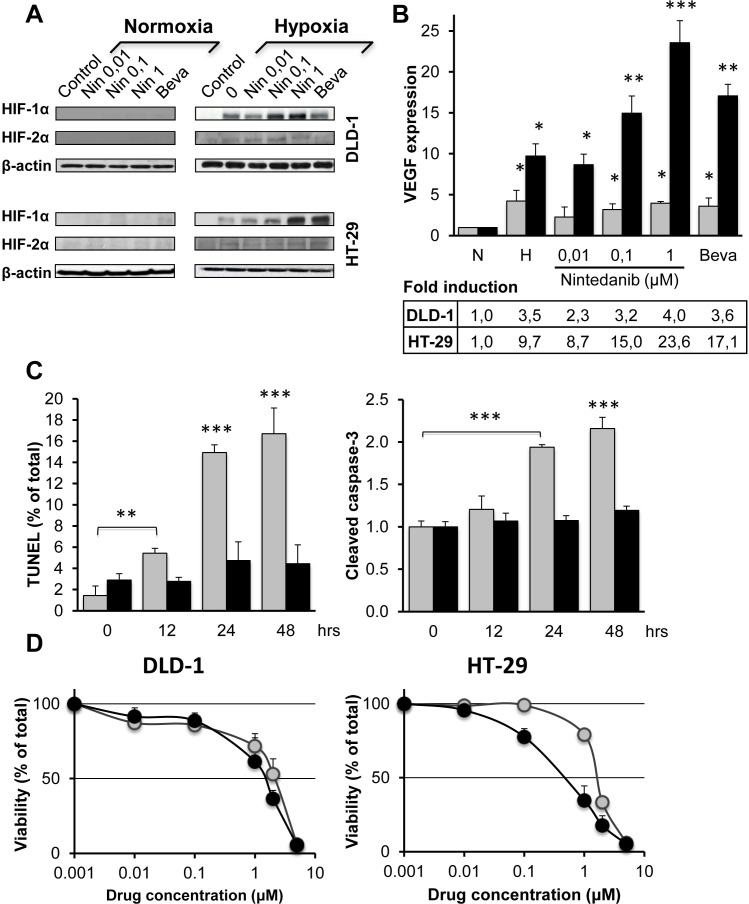
Influence of bevacizumab and nintedanib on CRC cells under normoxia and hypoxia (A) Western blot analysis of HIF-1alpha, HIF-2alpha and beta-actin in DLD-1 and HT-29 cells after 24 hrs exposure to nintedanib (0,01, 0,1 or 1 μM) or bevacizumab (250 μg/ml) under normoxia or hypoxia (1% O_2_). (B) Induction of VEGF mRNA in DLD-1 (grey columns) and HT-29 (black columns) cells following 24 hrs exposure to nintedanib (0,01, 0,1 or 1μM) or bevacizumab (250 μg/ml) under normoxia or hypoxia. VEGF expression was normalized with 36B4 mRNA and is the average of three independent experiments. The statistical analysis of experimental data was performed using a Student's paired t-test comparing the treatment group with the vehicle control. Bars, SD; * p < 0,05; ** p < 0,01; *** p < 0,001. (C) Hypoxia-induced apoptosis in DLD-1 (grey columns) and HT-29 (black columns) cells after hypoxia (1% O_2_) exposure for the indicated times as measured by the TUNEL assay (left) or by the formation of catalytically active cleaved caspase 3 (right). Values are the averages of at least two independent experiments done in duplicate. The statistical analysis of experimental data was performed using a Student's paired t-test comparing the treatment group with the vehicle control. Bars, SD; * p < 0,05; ** p < 0,01; *** p < 0,001. (D) Viability of DLD-1 cells (left) or HT-29 cells (right) after 24 hrs exposure to the indicated concentrations of nintedanib under normoxia (grey circles) or hypoxia (black circles) for 24 hrs followed by 96 hrs post-incubation under normoxia and MTT determination. Bars indicate SD and are shown when they exceed symbol size. The data represents three independent experiments each done in duplicate.

The marked differences in HIF expression between DLD-1 and HT-29 xenografts raise the question if DLD-1 cells are unable to activate HIF, unable to maintain HIF signaling, or rather if HIF signaling intensity in DLD-1 tumors is weak compared to HT-29 tumors. To answer this question, DLD-1 and HT-29 cells were exposed to acute hypoxia (1% O_2_) for 24 hrs and the expression of HIF-1alpha and HIF-2alpha was determined by Western blot analysis. The results show that the expression of the two HIF proteins is marginal under normoxia. In contrast, 24 hrs hypoxia is accompanied by HIF-1alpha induction in both cell lines whereas the expression of HIF-2alpha remains marginal (Figure 5A).

To establish if the HIF-1alpha protein was transcriptionally active, the expression of its downstream target, VEGF, was determined by qRT-PCR. Acute hypoxia was accompanied by a ~10-fold increase in VEGF expression for HT-29 cells, compared to the corresponding normoxic cells, whereas the corresponding increase in VEGF expression was ~3.5-fold for DLD-1 cells. Therefore, DLD-1 cells show a functional hypoxia response as indicated by upregulation of VEGF mRNA, although to a lesser degree than HT-29 (Figure 5B).

### Bevacizumab and nintedanib upregulate HIF-1alpha in both bevasizumab-sensitive and -resistant cells

We next explored if angiogenesis inhibitors might have a direct effect on HIF-VEGF signaling in CRC cells. Our results indicate that the presence of nintedanib or bevacizumab had no detectable effect on the expression of HIF-1alpha or HIF-2alpha under normoxia (Figure 5A). However, under hypoxia, nintedanib activated the expression of HIF-1alpha in a dose-dependent manner in both cell lines (2 to 3-fold, compared to the normoxia control) (Figure 5A). In addition, bevacizumab was able to induce HIF-1alpha almost 4-fold in the HT-29 cells whereas its influence was marginal in DLD-1 cells (Figure 5A).

The drug-induced increase of HIF-1alpha protein had modest influence on the expression of VEGF mRNA in hypoxic DLD-1 cells (Figure 5B). In clear contrast, for hypoxic HT-29 cells, nintedanib-exposure was accompanied by a dose-dependent increase in mRNA VEGF expression that reached ~2,4 times the levels of the untreated controls, whereas bevacizumab increased mRNA VEGF expression ~1.8-fold. Therefore, although drug exposure was associated with increased HIF-1alpha accumulation under short-term hypoxia in both cell lines, VEGF mRNA expression was only increased for HT-29 cells.

### Bevacizumab-resistant cells show increased hypoxia tolerance

Next, DLD-1 and HT-29 cells were exposed to hypoxia for up to 48 hrs and the fraction of apoptotic cells was determined by the TUNEL assay (Figure 5C left). The results show that hypoxia exposure was accompanied by rapid apoptosis in DLD-1 cells to reach almost 17% apoptotic cells by 48 hrs, whereas the level of apoptosis stayed below 5% for HT-29. These findings were confirmed by a caspase 3 assay that measures the formation of catalytically active cleaved caspase 3 (Figure 5C right).

### Nintedanib shows increased toxicity toward bevacizumab-resistant cells under hypoxia

To determine the influence of hypoxia on nintedanib toxicity, cells were exposed to different doses of nintedanib for 24 hrs under hypoxia or normoxia followed by 96 hrs post-exposure under normoxia and MTT determination. The results show that nintedanib is slightly (~30%) more toxic for DLD-1 cells under hypoxia compared to normoxia (Figure 5D left). In comparison, nintedanib was almost 3 times more toxic to HT-29 cells under hypoxia, suggesting that nintedanib attenuates the survival signaling that usually protects HT-29 from hypoxia-mediated cell death (Figure 5D right).

## DISCUSSION

Bevacizumab shows excellent activity in a subset of mCRC patients while others never respond. A key question is if bevacizumab-resistance is limited to this particular agent or rather is indicative of a general resistance to angiogenesis inhibition. To clarify this question, we identified the HT-29 xenograft model as naturally bevacizumab-resistant in contrast to DLD-1 xenografts that are bevacizumab-sensitive. Prolonged (28 days) exposure to the angiokinase inhibitor nintedanib showed that DLD-1 and HT-29 xenografts are both sensitive to nintedanib at doses with no detectable toxic side effects. These findings suggest that the antitumor activity of at least some small molecule angiokinase inhibitors is not limited by the mechanisms underlying natural bevacizumab resistance.

Next, we characterized the differences between bevacizumab-sensitive and -resistant cells, with special attention to VEGF signaling. Comparison of the vascular density in DLD-1 and HT-29 tumors by IHC analysis reveals that bevacizumab treatment is associated with important vascular pruning in the sensitive DLD-1, but not in the resistant HT-29 xenografts. In clear contrast, nintedanib showed comparable effects on the microvascular density in both xenograft models. To establish if the intrinsic bevacizumab resistance of HT-29 xenografts might be linked to elevated levels of stroma-derived VEGF, the concentrations of human and murine VEGF were determined. The results show that for both tumor types, murine VEGF represents, at the most, 1% of the total amount of VEGF and is therefore unlikely to influence the response to angiogenesis inhibition. Interestingly, HT-29 tumors express at least four times more human VEGF, compared to the DLD-1 tumors, which likely contributes to the bevacizumab-resistant phenotype.

To establish the mechanistic basis for tumor growth inhibition, the levels of DNA synthesis, apoptosis and necrosis were determined. The results show that prolonged exposure to the two drugs resulted in a mixture of cytostatic and cytotoxic effects, independent of the tumor model. However, nintedanib exposure was associated with significantly more apoptosis in HT-29 than in DLD-1 xenografts, suggesting that in this tumor model, nintedanib may not only act on the microvasculature, but also attenuate the survival response of the tumor cells to environmental stress.

**Figure 6:**
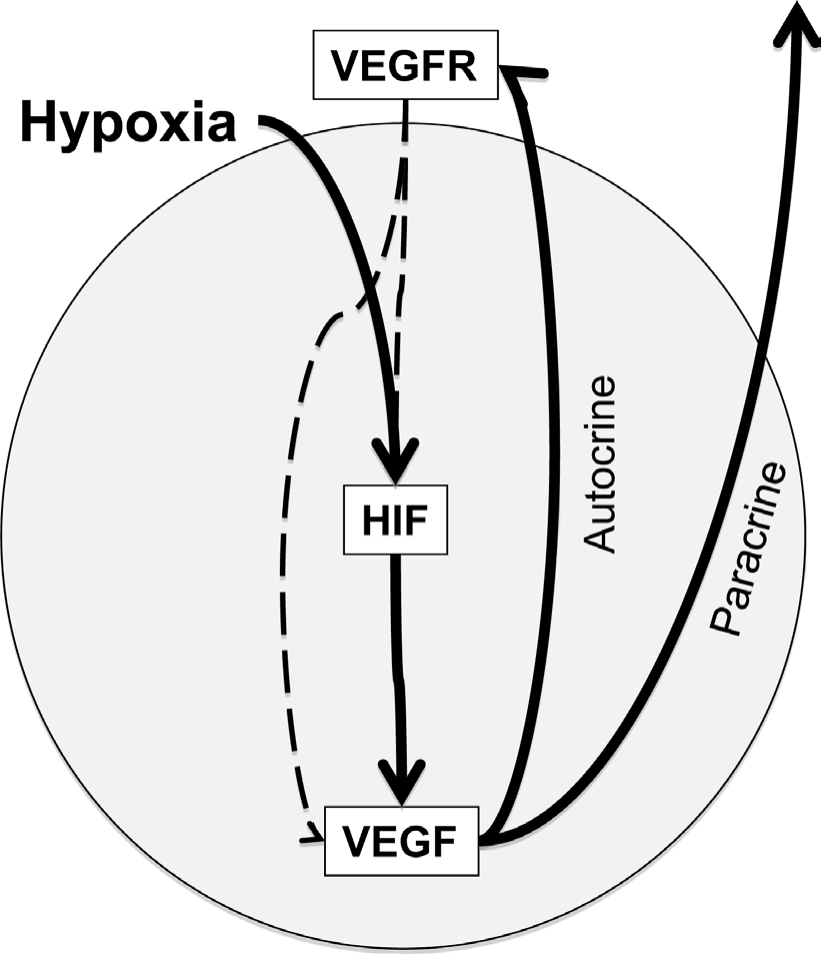
HIF-VEGF-VEGFR signaling in CRC cells Hypoxia exposure activates HIF, thereby increasing the expression of VEGF, a HIF-transcriptional target. Subsequent binding of VEGF to its receptor on either CRC cells (autocrine pathway) or tumor-associated endothelial cells (paracrine pathway) leads to activation of VEGFR and its downstream signaling pathways. Receptor tyrosine kinases like VEGFR are able to activate and/or stabilize HIF and VEGF, thereby promoting a positive feed-back loop.

To characterize the signaling intensity of the HIF-VEGF signaling pathway (summarized in Figure 6), the expression of HIF-1alpha, HIF-2alpha, VEGF and the active, autophosphorylated forms of VEGFR1 and VEGFR2 was determined by IHC followed by quantitative image analysis. The results revealed striking differences between the two tumor models. Drug treatment was accompanied by a general downregulation of all signaling components of the HIF-VEGF-VEGFR autocrine loop in DLD-1 tumors, in clear contrast to HT-29 tumors, where the same pathway was upregulated. Interestingly, for HT-29 tumors, treatment with bevacizumab was accompanied by much higher activation of autocrine VEGF signaling than nintedanib, thereby identifying a key difference between the two agents.

mTORC1 integrates multiple signaling pathways downstream of activated receptor tyrosine kinases. Accordingly, recent findings suggest that the degree of mTORC1 activity, as determined by the levels of the Ser240/Ser244 phosphorylated form of the ribosomal S6 protein (pS6), serve as a robust biomarker for the antitumor activity of at least some signaling pathways inhibitors. Quantitative IHC analysis showed that treatment with both bevacizumab and nintedanib was accompanied by downregulation of mTORC1 activity in DLD-1 tumors. In clear contrast, bevacizumab exposure was associated with upregulation of mTORC1 activity in HT-29 tumors whereas nintedanib treatment was associated with a marked decrease of mTORC1 activity, compared to the vehicle control. For the bevacizumab-treated HT-29 tumors, the increase in mTORC1 activity is fully coherent with the elevated levels of active, phosphorylated VEGFR1 and VEGFR2. In contrast, the strong downregulation of mTORC1 in the nintedanib-treated HT-29 tumors was unexpected. The downregulation is unlikely to rely on VEGF-signaling alone, but likely involves other nintedanib targets like FGFR (fibroblast growth factor receptor) and PDGF (platelet-derived growth factor receptor receptor) family members [5].

Another unexpected finding was that HIF-1alpha was upregulated in the nintedanib-treated HT-29 tumors in spite of the marked downregulation of mTORC1, a well-established negative regulator of HIF-1alpha translation. The upregulation of HIF-1alpha is coherent with the loss of tumor-associated microvessels that results in tumor hypoxia and activation of autocrine VEGF signaling. Furthermore, it should be noted that the regulation of HIF-1alpha is complex and is mediated at several levels including transcription, translation as well as protein stability. Even on the translational level, our previous results suggest that HIF-1alpha expression can be modulated by (at least) two different agents, rapamycin via mTORC1, and irinotecan, that acts in a mTORC1-independent manner [33].

Interestingly, the beva-resistance of the HT-29 xenografts could be overcome by combining bevacizumab with low dose nintedanib. The additional antitumor effect was characterized by a diminution, rather than an increase, of mTORC1 activity as well as by increased inhibition of the tumor microvasculature.

A crucial question is if bevacizumab-sensitive cells are unable to activate HIF-signaling in response to hypoxia, unable to maintain HIF activation, or if the HIF signaling pathway is functional, but less prominent compared to bevacizumab-resistant cells. To answer this question, DLD-1 and HT-29 cells were incubated under hypoxia for 24 hrs and the induction of HIF-1alpha and HIF-2alpha was determined. The results show that both cell lines express little, if any, HIF-1alpha or HIF-2alpha under normoxia. In contrast, hypoxia is accompanied by robust HIF-1alpha induction in both cell lines whereas the induction of HIF-2alpha is marginal. These findings are coherent with previous work reporting that HIF-1alpha drives the initial response to hypoxia whereas HIF-2alpha is needed for the chronic response [34]. Next, we determined the capacity of HIF to increase the expression of VEGF mRNA. The results reveal that although VEGF was induced by hypoxia in both cell lines, the increase in VEGF levels was more prominent for HT-29 cells (~10-fold increase) than for DLD-1 cells (~3-fold increase). Interestingly, the differences in VEGF expression *in vitro* were comparable to the differences in VEGF expression observed for the corresponding xenografts. Therefore, the *in vivo* differences in basal VEGF levels appear to be a result of an intrinsically higher HIF-VEGF signaling intensity in bevacizumab-resistant cells.

Unexpectedly, the hypoxia-mediated up-regulation of HIF-1alpha protein was further increased in the presence of nintedanib and bevacizumab, suggesting that the two agents can influence tumor cells directly under cellular stress. The activity of nintedanib is consistent with previous findings, where nintedanib was shown to modulate cell cycle progression and viability of a wide range of CRC cell lines [35]. The influence of bevacizumab is more unexpected considering that bevacizumab is a ligand-targeted agent that is unable to enter the tumor cells. However, two recent articles report that chronic *in vitro* exposure of CRC cells to bevacizumab was accompanied by the emergence of a more migratory and invasive phenotype [36, 37], which is coherent with a direct effect of bevacizumab on the tumor cells, as observed here.

HT-29 cells were more resistant to hypoxia-induced apoptosis compared to DLD-1 cells consistent with their bevacizumab-resistant phenotype *in vivo*. If the increased survival of HT-29 cells under hypoxia depends on their potent kinase activity including autocrine VEGF-signaling, we would predict that nintedanib should be more cytotoxic under hypoxic conditions than under normoxia. In contrast, we would expect a smaller difference for DLD-1 cells that are inherently hypoxia-sensitive and appear to have weaker angiokinase signaling. In agreement, the results showed almost three-fold increased activity of nintedanib under hypoxia in HT-29 cells, whereas nintedanib was only marginally more active under hypoxia toward DLD-1 cells. The increased activity of nintedanib under hypoxia is a noticeable feature, since other tyrosine kinase inhibitors like gefitinib have been reported to show less, rather than more, cytotoxic activity under hypoxia [38].

Taken together, our data indicate that intrinsic bevacizumab-resistance is multifactorial and associated with i) the high levels of VEGF in the tumor environment that renders the endothelial cells more resistant to bevacizumab, ii) strong VEGFR1 and VEGFR2 signaling in the tumor cells that leads to mTORC1 activation as well as iii) resistance to hypoxia-induced apoptosis. We further show that all these resistance mechanisms can be overcome by nintedanib. These findings suggest that the antitumor activity of at least some small molecule angiokinase inhibitors is not limited by the mechanisms underlying natural bevacizumab resistance and provide a rational for clinical trials of nintedanib in CRC patients that do not respond to bevacizumab, alone or in combination with bevacizumab.

## MATERIALS and METHODS

### Xenograft models

The antitumor activity of bevacizumab and nintedanib was evaluated in athymic mice (female NMRI-Fox 1nu, 6 weeks old) from Taconic (Skensved, Denmark) bearing DLD-1 or HT-29 CRC xenografts. Two (HT-29) or five (DLD-1) million cells were injected into the right flank, and the treatments were started when the tumors were palpable (median tumor volume ~100 mm^3^). The animals were weighted daily and the tumor size was determined three times per week. Tumor volumes (mm^3^) were calculated according to formula: [(length^2^; × width)/2]. Treated/Control (T/C) values were calculated as follows: average tumor volume of treated animals/average tumor volume of control animals x100. Each treatment group was composed of at least 7 animals. Animals were treated according to institutional guidelines.

### Immunohistochemistry

Biomarker analysis was carried out with tumors collected after 28 days of treatment. To measure *in vivo* DNA synthesis, the thymidine analog 5-ethynyl-2’-deoxyuridine (EdU, Life Technologies) was administered 48 hrs before sacrifice (500 μg *i.p.*). The incorporated EdU was revealed by a fluorescent-azide coupling reaction (Click-iT # C10337, Life Technologies) of paraffin-embedded tumor samples and counterstained by DAPI to reveal the nuclei of individual cells. The proportion of apoptotic tumor cells was scored by the TUNEL assay (In Situ Cell Death Detection kit, Roche Applied Science # 11684795910).

The following antibodies were used for immunohistochemistry (IHC) analysis: anti-CD31 (BD Bioscience # 550274), anti-HIF-1alpha (BD Bioscience # 610958), anti-HIF-2alpha (Novus Biological NB-100122), anti-VEGF (Santa Cruz # sc-152), anti-pVEGFR1 (Millipore 07-758) that recognizes phospho-Tyr1213 VEGFR1, anti-pVEGFR2 (Santa Cruz # sc-101819) that recognizes phospho-Tyr1175 VEGFR2, and anti-pS6 (Cell Signaling # 2211) that recognizes phospho-Ser240/244 S6. The relevant Cy3-conjugated secondary antibodies were obtained from Jackson ImmunoResearch. All images were captured by a fluorescence microscope and the fluorescence intensities were determined by the MetaMorph software (Universal Imaging Corporation) for quantitative image analysis.

Blood vessel density is expressed as the CD31-positive area, in % of total, and represents the averages of at least 6 fields/tumor for at least 3 different tumors. DNA synthesis is determined as the ratio between EdU-positive cells and the total number of viable cells and is the average of 6 fields/tumor (each field representing approximately 1700 cells) from at least 3 different tumors. Apoptosis is expressed as the % area of TUNEL-positive cells compared to the total area of viable cells, and is the average of 6 fields/tumor for at least 3 different tumors. For necrosis, the data indicates the surface of necrotic cells as % of the total surface and is the average of 6 fields/tumor for at least 3 different tumors.

For the quantitative analysis of the signal intensity for HIF-1alpha, HIF-2alpha, VEGF, pVEGFR1, pVEGFR2 and pS6, the data represents the average fluorescence intensity of treated tumors compared to the treatment intensity of control tumors and is the average of 6 fields/tumor for at least 3 different tumors.

### ELISA assay for VEGF

Healthy tumor tissues were collected from untreated, frozen tumors (three tumors per xenograft model), and protein extracts were prepared in RIPA buffer according to the manufacturer's instructions. VEGF levels were determined by Quantikine ELISA (R&D System # DVE00 (human, tumor-derived VEGF) and MMV00 (murine, stroma-derived VEGF). The values represent the average of 3 independent experiments, each done in duplicate.

### Tumor cells, apoptosis and viability assays

HT-29 cells were kindly provided by Richard Camalier, Division of Cancer Treatment and Diagnosis tumor repository (NCI, US) whereas DLD-1 cells were a generous gift from Richard Hamelin (Saint-Antoine Research Center, Paris, France).

To determine the induction of hypoxia-induced apoptosis, HT-29 and DLD-1 cells were seeded on coverslips in DMEM medium with 5% fetal calf serum under hypoxia (1% O_2_). Cover slips were removed after 0, 12, 24 and 48 hrs hypoxia and subjected to TUNEL analysis. Alternatively, hypoxia-induced apoptosis was determined by the capacity of catalytically active cleaved caspase-3 to generate fluorescent reaction products (Caspase 3 fluorescence assay kit, Cayman chemical, #10009135).

Cellular viability was determined by the MTT (methylthiazolyldiphenyl-tetrazolium bromide) viability assay as described previously [39] with minor modifications. Cells were incubated with different concentrations of nintedanib under normoxia or hypoxia (1% O_2_) for 24 hrs followed by 96 hrs post-incubation under normoxia and MTT determination.

### Real-time RT (reverse transcription)-PCR and Western blot analysis

Total RNA was extracted from CRC cells using the TRIzol® RNA purification reagent. RNA quantity and purity were determined by using a NanoDrop ND-1000. Total RNA (1 μg) from each sample was reverse transcribed and real-time RT-PCR measurements were performed as described previously [40, 41] using a Mx3000P apparatus (Agilent) with the corresponding SYBR Green kit. PCR primers were designed with Primer 3 (Agilent) as follows: VEGF, upper, 5’-CGAAGTGGTGAAGTTCATGGATG-3’, lower, 5’-TTCTGTATCAGTC TTTCCTGGTGAG. 36B4 (also known as RPLP0), upper, 5’-GATTGGCTACCCAACTGTTG-3’; lower, 5’-CAGGGGCAGCAGCCAC AAA-3’. Gene expression was normalized to 36B4.

Western blot analysis was carried out as described previously [42]. The primary antibodies were directed against HIF-1alpha (BD Bioscience # 610958), HIF-2alpha (Novus Biological # NB-100122) or beta-actin (Santa Cruz # sc-47778), while the corresponding secondary antibodies were purchased from Jackson ImmunoResearch.

### Statistical analysis

The statistical analysis of experimental data was performed using a Student's paired *t*-test, and results are presented as mean ± standard deviation (SD).
